# Prediction of Arteriovenous Access Dysfunction by Mel Spectrogram-based Deep Learning Model

**DOI:** 10.7150/ijms.98421

**Published:** 2024-08-19

**Authors:** Tung-Ling Chung, Yi-Hsueh Liu, Pei-Yu Wu, Jiun-Chi Huang, Yi-Chun Tsai, Yu-Chen Wang, Shan-Pin Pan, Ya-Ling Hsu, Szu-Chia Chen

**Affiliations:** 1 Graduate Institute of Medicine, College of Medicine, Kaohsiung Medical University, Kaohsiung, Taiwan.; 2 Division of Nephrology, Department of Internal Medicine, Kaohsiung Veterans General Hospital, Kaohsiung, Taiwan.; 3 Graduate Institute of Clinical Medicine, College of Medicine, Kaohsiung Medical University, Kaohsiung, Taiwan.; 4 Department of Internal Medicine, Kaohsiung Municipal Siaogang Hospital, Kaohsiung Medical University Hospital, Kaohsiung Medical University, Kaohsiung, Taiwan.; 5 Division of Cardiology, Department of Internal Medicine, Kaohsiung Medical University Hospital, Kaohsiung Medical University, Kaohsiung, Taiwan.; 6 Division of Nephrology, Department of Internal Medicine, Kaohsiung Medical University Hospital, Kaohsiung Medical University, Kaohsiung, Taiwan.; 7 Division of General Medicine, Kaohsiung Medical University Hospital, Kaohsiung Medical University, Kaohsiung, Taiwan.; 8 Faculty of Medicine, College of Medicine, Kaohsiung Medical University, Kaohsiung, Taiwan.; 9 Muen Biomedical and Optoelectronics Technologies Inc., Taipei, Taiwan.

**Keywords:** arteriovenous access dysfunction, deep learning model, electronic stethoscope, Mel spectrograms

## Abstract

**Background:** The early detection of arteriovenous (AV) access dysfunction is crucial for maintaining the patency of vascular access. This study aimed to use deep learning to predict AV access malfunction necessitating further vascular management.

**Methods:** This prospective cohort study enrolled prevalent hemodialysis (HD) patients with an AV fistula or AV graft from a single HD center. Their AV access bruit sounds were recorded weekly using an electronic stethoscope from three different sites (arterial needle site, venous needle site, and the midpoint between the arterial and venous needle sites) before HD sessions. The audio signals were converted to Mel spectrograms using Fourier transformation and utilized to develop deep learning models. Three deep learning models, (1) Convolutional Neural Network (CNN), (2) Convolutional Recurrent Neural Network (CRNN), and (3) Vision Transformers-Gate Recurrent Unit (ViT-GRU), were trained and compared to predict the likelihood of dysfunctional AV access.

**Results**: Total 437 audio recordings were obtained from 84 patients. The CNN model outperformed the other models in the test set, with an F1 score of 0.7037 and area under the receiver operating characteristic curve (AUROC) of 0.7112. The Vit-GRU model had high performance in out-of-fold predictions, with an F1 score of 0.7131 and AUROC of 0.7745, but low generalization ability in the test set, with an F1 score of 0.5225 and AUROC of 0.5977.

**Conclusions:** The CNN model based on Mel spectrograms could predict malfunctioning AV access requiring vascular intervention within 10 days. This approach could serve as a useful screening tool for high-risk AV access.

## Introduction

In patients undergoing hemodialysis (HD), a well-functioning arteriovenous (AV) access is essential to provide sufficient blood flow for dialysis adequacy. Dysfunctional AV access may result in reduced or interrupted blood flow during HD sessions. Previous studies have shown that AV access dysfunction is associated with increased hospitalization rates, morbidity, and financial burdens on medical care in HD populations 1-3. Monitoring the blood flow of AV access and timely interventions for AV access dysfunction are crucial to prevent the loss of the AV access 4.

The current international guidelines recommend regular physical examinations of an AV fistula (AVF) or AV graft (AVG) by trained healthcare practitioners to identify clinical signs of dysfunctional AV access 5. Notable clinical indicators suggesting AV access dysfunction include ipsilateral edema, alterations in pulse, abnormal thrill and bruit 5. Auscultation is a clinical monitoring strategy used to detect abnormal blood flow in AV access 6, and abnormal bruit (high pitched with a systolic component in the area of stenosis) may indicate stenosis of AV access 6, 7. On the other hand, Duplex ultrasound and angiography provide more reliable methods for assessing blood flow in AV access compared to a physical examination 8. However, the accuracy of ultrasound depends on the skill of the operator, and angiography is an invasive procedure. Therefore, there is a need for a reliable, non-invasive, point-of-care tool to evaluate the functionality of AV access.

The Mel Spectrogram is a two-dimensional representation of the audio recording and is widely used in audio recognition tasks. It can extract features from audio signals and applies a frequency-domain filter bank to audio signals that are windowed in time. By filtering background noise, it enables the deep learning to extract physiological and pathological features from the audio signals, thereby enhancing classification accuracy 9. Many studies have proposed Mel spectrogram-based deep learning models for heart sounds classification and have demonstrated high performance 10-12. Regarding the application of Mel spectrogram in AV access, several studies demonstrated the feasibility of deep learning to analyze Mel spectrogram which were generated from AV access bruit sound. Ota et al used a Convolutional Neural Network (CNN) model to classify AV bruit sound to multiple categories 13, while Park et al developed deep CNN model to predict the presence of significant (≥50%) AVF stenosis 14 and 6-month primary patency 15. These studies use CNN to predict AV access dysfunction or to classify AV access sounds and show outstanding results. However, the performance of different deep learning models has not been compared in these two studies. On the other hand, Zhou et al. found that a vision transformer trained on spectrogram images of AVF blood flow sounds can achieve the best performance in screening for AVF stenosis, comparable to a physical exam by a nephrologist 16.

In this study, we utilized an electronic stethoscope to record bruits in AV access and converted the signals to Mel-scaled spectrograms. We developed three different deep learning methods—Convolutional Neural Network (CNN), Convolutional Recurrent Neural Network (CRNN), and Vision Transformers-Gate Recurrent Unit (ViT-GRU)—to analyze and classify the data from the spectrograms and predict the potential need for vascular interventions for AV access within 10 days. In addition, we compared the performance of these three deep learning models to find the most feasible model for clinical use. The aim of this study was to develop a useful and point-of-care tool for screening high-risk AV access in HD patients.

## Materials and Methods

### Study population and study design

This prospective study was conducted at a single HD center from May 2021 to July 2022. Patients were included if they were: (1) age > 20 years; (2) with an AVF or AVG as vascular access for dialysis; and (3) under prevalent HD treatment for at least 90 days. The exclusion criteria were: (1) age ≤ 20 years; (2) patients with an acute illness; and (3) hospitalized patients. Baseline characteristics of the patients were obtained from their electronic medical records. The study was approved by the Institutional Review Board of Kaohsiung Medical University Hospital (KMUHIRB-E(l)-20210092). Informed consent was obtained from all of the participants.

**Figure 1:**
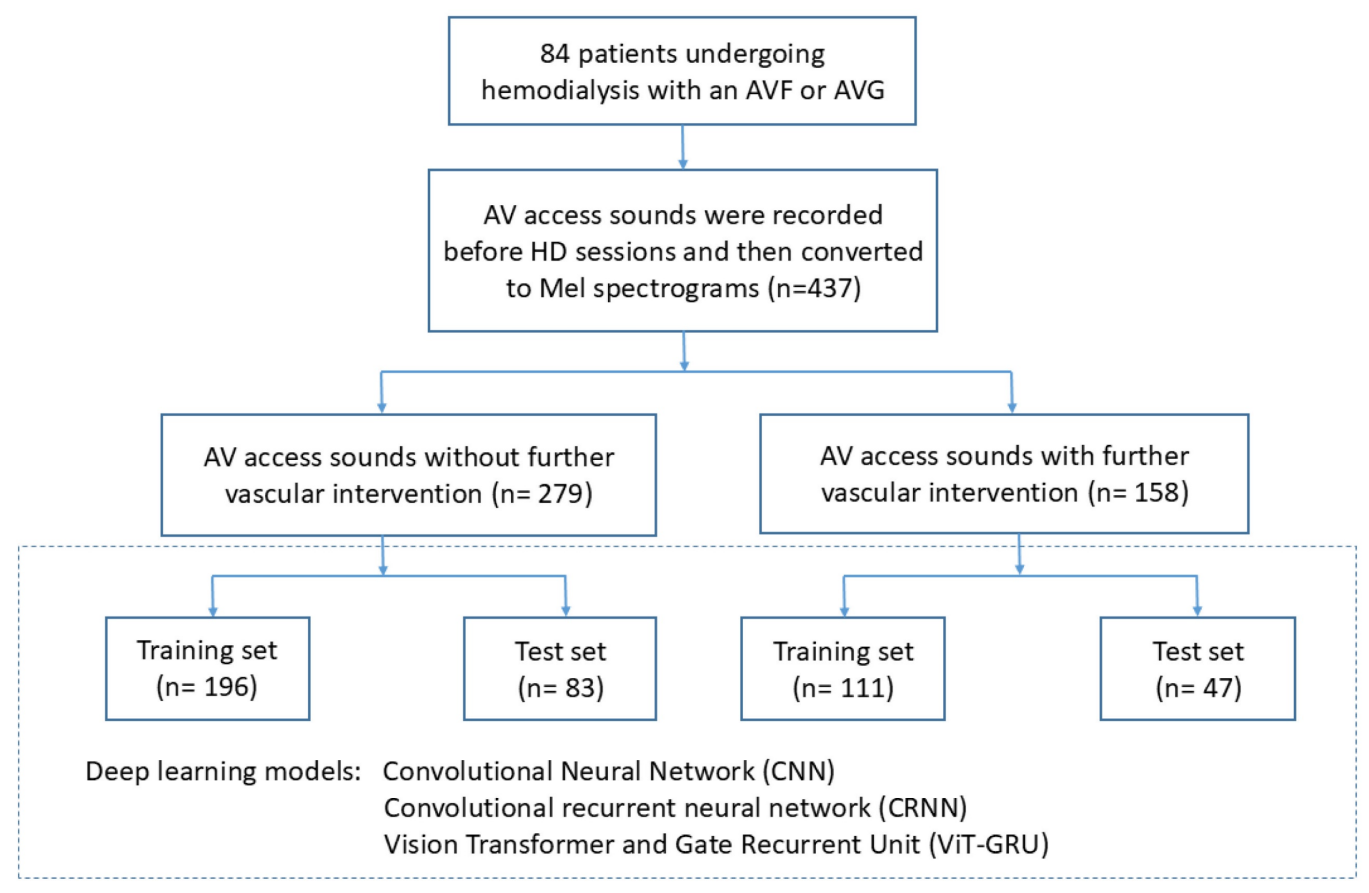
Study flow chart.

The flowchart of this study is shown in Figure 1. The patients who received a vascular intervention were classified as the “vascular intervention group”, and their recordings obtained within 10 days before the vascular intervention were extracted and classified as “vascular intervention recordings”. The patients who did not receive a vascular intervention were classified as the “non-vascular intervention group”, and all of their recordings were classified as “non-vascular intervention recordings”. Eighteen randomly selected patients from both the vascular intervention and non-vascular intervention groups were used for the test set, and the remaining patients served as the training set for cross-validation. Table 1 illustrates the distribution of patients and audio recordings between the training and test sets.

The definition of AV access dysfunction was a decrease in blood flow or complete cessation of blood flow in the AV access, as detected by physical examination or resulting in the disruption of hemodialysis therapy. Vascular intervention was defined as a dysfunctional AV access that received either surgical intervention (such as thrombectomy, intraoperative percutaneous transluminal angioplasty, and anastomosis revision) or endovascular intervention (such as mechanical or pharmacological thrombolysis with angioplasty).

### Recording of AV access sounds

AV access sounds were recorded weekly before HD sessions using an electronic stethoscope (AccurSound AS101, Heroic Faith Medical Science Co., Ltd., Taipei, Taiwan) from three different sites: the arterial needle site (A), the venous needle site (V), and the midpoint between the arterial and venous needle sites (M). The audio signals were converted to Mel spectrograms using Fourier transformation with Airmod software (Heroic Faith Medical Science Co., Ltd., Taipei, Taiwan) and then utilized to develop deep learning models.

### Data preprocessing

The audio recordings were first reduced using a spectral gating method which transformed the signals to spectrograms and calculated the frequency-varying thresholds for each frequency band 17, 18. The thresholds were used to filter noises below the frequency-varying thresholds. After noise reduction, all recordings were fixed to a length of 75000 samples (18.75 seconds at a sampling rate of 4000 Hz). This length was chosen as it was the maximum length of all recordings in the dataset. Recordings with a length below 75000 were padded with 0 from the recording end. Mel-scaled spectrograms were computed after padding, with the number of Mel-frequency cepstral coefficients set at 40. The Mel spectrograms were normalized based on local cepstral mean and variance calculated in sliding windows 19.

### Development of deep learning models

Deep learning models were developed using a 3-fold cross-validation procedure. Out-of-fold predictions and an additional test set were used to evaluate model performance. The predicted results of the test set were obtained by averaging the output from each model.

Three deep learning models with different architectures were developed and compared: (1) CNN, (2) CRNN, and (3) ViT-GRU. The shape of the Mel spectrograms was 40 x 369, where 40 is the number of Mel-frequency cepstral coefficients, and 369 is the number of time series. Three channels of the Mel spectrograms which were processed from A (arterial needle site), V (venous needle site) and M (midpoint between the A and V) positions were then concatenated and input into the deep learning models. When training the models, an Adam optimizer 19 with a learning rate of 3×10^(-4) was used, and the loss function was binary cross-entropy. The batch size was set to 32, and the number of training epochs was 300.

The first proposed model was a CNN model, which was composed of a convolutional layer, a pooling layer, and a fully connected layer. The convolutional layer was used to extract feature maps from the Mel spectrograms, and the pooling layer decreased the spatial size of the feature maps. The feature maps were then flattened and passed through the fully connected layer, and a sigmoid activation function was used to obtain the probabilities as the final output. Table 2 shows the architecture of the CNN model.

The proposed CRNN model combined the CNN model with a recurrent neural network (RNN). RNN models can help extract temporal features from time series data by retaining the information of the previous time step. In this CRNN model, a bidirectional gated recurrent unit (GRU) 20, 21 with 128 units was combined with the CNN model. In the bidirectional architecture 20, the output of two RNN layers was concatenated. One RNN layer operated in a forward direction, and the other RNN layer operated in a backward direction. With this bidirectional architecture, the model could obtain information from the previous and next time steps simultaneously. In a GRU unit 21 there are two gates: an update gate and a reset gate. The reset gate decides the amount of information to retain from the previous step. The update gate controls the proportion of information taken from the previous and current steps and passes it to the next step. Table 3 summarizes the architecture of the CRNN model.

The final proposed mode was the ViT-GRU model 21, 22, which combined a vision transformer (ViT) model and a bidirectional GRU model. A ViT model 22 applies a transformer, a self-attention architecture, to 2-dimentional data. It can split 2-dimentional data into several patches and applies linear projection in order to embed patches. The embedded patches are then provided as a sequence to transformer encoders. The ViT part of the proposed ViT-GRU model had two layers of transformer encoders, including a multi-headed self-attention (MSA) 23 block and a multi-layer perceptron (MLP) 24 block. Layer normalization 25 was applied before each block, and the residual connections were used to add the input and output of each block. In the MSA block 23, eight self-attention heads were run in parallel, and their output was linearly combined. Each attention mechanism (head) comprised three components: the queries, keys, and values. These three components were all obtained from the linear transformations of the input. First, the self-attention scores were computed by multiplying (dot product) the queries with the keys; this represented the concept of self-attention, because both the queries and keys were the linear transformations of the input. Self-attention scores were then passed through a Softmax function to generate the attention weights. These weights were used to modify the values, so the input data were modified with the attention weights. The MLP block 24 included two layers of fully connected layers with a Gaussian error linear unit (GELU) 26 activation function. The GRU part of the ViT-GRU model functioned the same way as in the CRNN model. A dropout layer and two fully connected layers were added at the end of the model, and a sigmoid activation function was used to obtain the probabilities. Table 4 outlines the architecture of the ViT-GRU model.

## Results

### The characteristics of study population

A total of 84 hemodialysis patients with 437 audio recordings were collected. The median age of these patients is 64.0 years, with 42 males and 42 females. Among all the patients, 75 patients were using an AVF (89.3%), while 9 patients were using an AVG (10.7%). During the follow-up period, AV access dysfunction occurred in 29 patients (34.5%), with 23 using AVF (79.3%) and 6 using AVG (20.7%).

### Performance of the deep learning models

**Figure 2:**
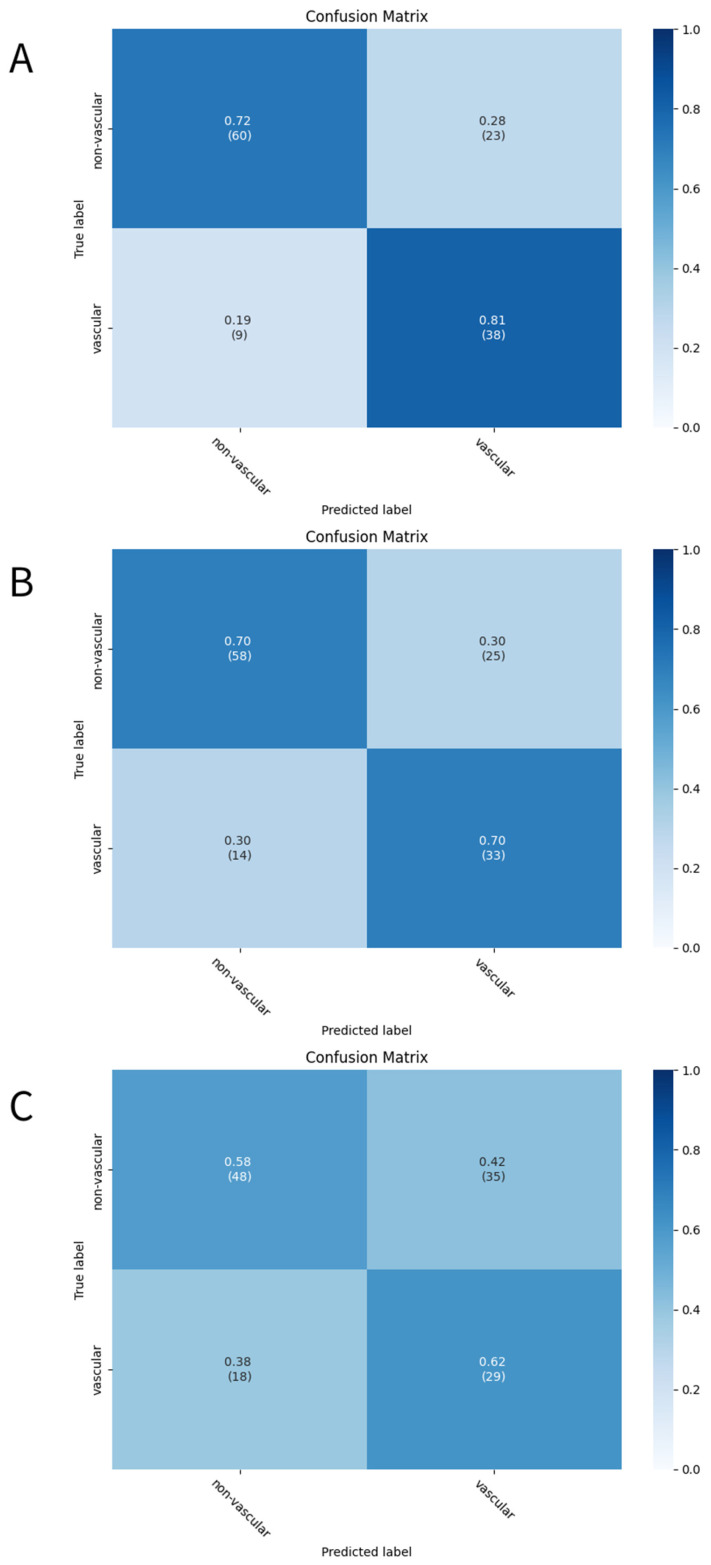
**The confusion matrixes of the deep learning models.** The confusion matrixes of (A)CNN, (B)CRNN, and (C)ViT-GRU models in the test set. The x-axis and y-axis in the confusion matrixes represent the predicted labels and the true labels, respectively.

**Figure 3:**
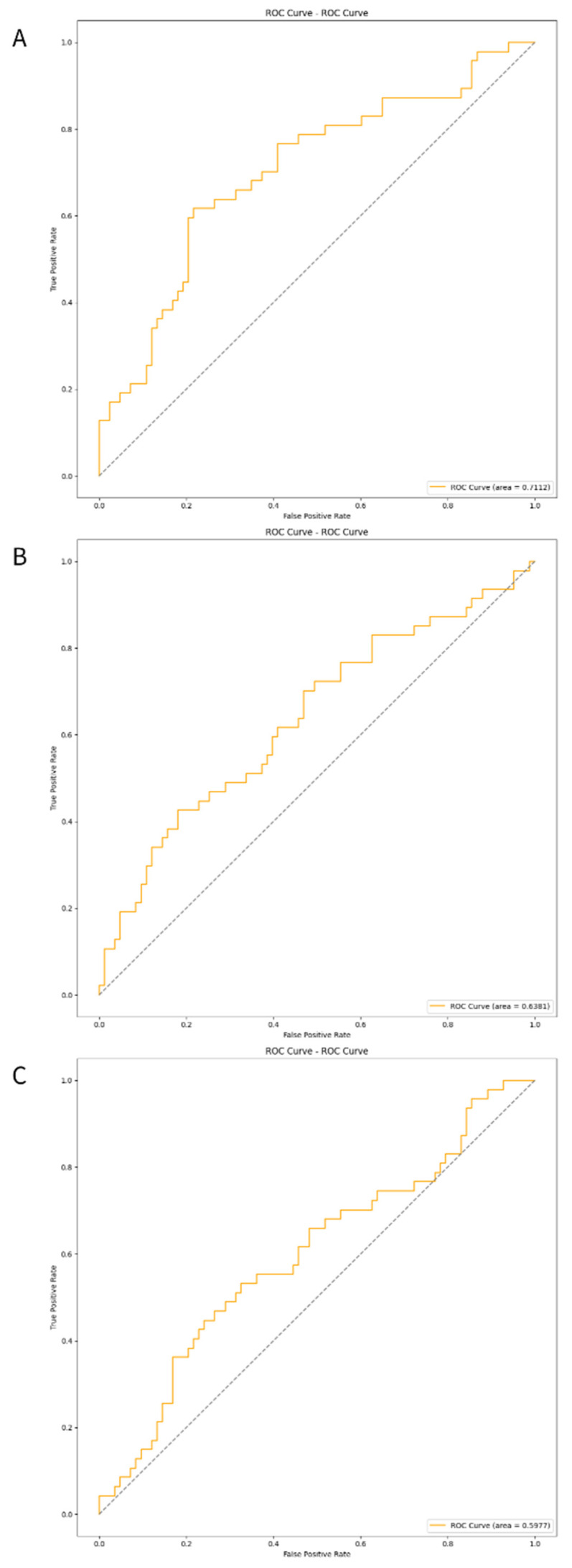
** The ROC curves of the deep learning models in test set.** The ROC curves of (A)CNN, (B)CRNN, and (C) ViT-GRU models in the test set. The orange line showing the ROC curve of the model, and the blue line is a diagonal line.

The three developed deep learning models (CNN, CRNN, and ViT-GRU) were used to predict AV access dysfunction. The results of out-of-fold predictions of these three models are listed in Table 5, and the performance in the test set is shown in Table 6. The confusion matrixes of the three models in the test set are illustrated in Figure 2. The areas under the receiver operating characteristic curves (AUROCs) for the CNN, CRNN, and ViT-GRU models on the test set were 0.7112, 0.6381, and 0.5977, respectively, as shown in Table 6 and Figure 3.

In out-of-fold predictions, the ViT-GRU model achieved the highest performance with an F1 score of 0.7131 and AUROC of 0.7745. However, when evaluating the model performance using an additional test set, the ViT-GRU model had the worst performance with an F1 score of 0.5225 and AUROC of 0.5977 (Table 6). This showed the low generalization ability of our ViT-GRU model. On the other hand, the CNN model showed more stable performance, with an AUROC > 0.7 in both out-of-fold predictions and in the test set.

## Discussion

In this study, we developed three different deep learnings models to analyze Mel-scaled spectrograms of AV access sounds from HD patients and then compared their performance on predicting AV access dysfunction that would necessitate a vascular intervention within 10 days. Among these three distinct deep learning models, the CNN model exhibited the most consistent diagnostic performance in the test set. Our results suggest that a deep learning model is a feasible method for analyzing AV access sounds.

Given that stenosis can occur at any site within the AV access, we recorded AV access sounds using an electronic stethoscope from three different sites (arterial needle site, venous needle site, and the midpoint between the two sites) instead of a single site, aiming to generate a more comprehensive audio dataset. Following conversion of the audio files into visual images, the deep learning models could extract features, classify patterns, and subsequently interpret the results of the Mel spectrograms from malfunctioning AV accesses. In this study, we compared the performance of three distinct deep learning models: CNN, CRNN, and ViT-GRU. These models represent the state-of-the-art in time-series image classification 27, 28. CNNs are one of the most popular deep learning networks 28 and are widely used in medical image classification due to their self-learning ability to recognize lesion areas from pixel-level features. For example, CNNs have been used for automatic lung pattern classification to diagnose interstitial lung diseases from computed tomography scans 29, detect melanoma by analyzing clinical images 30, and detect mammography masses and classify them into benign and malignant lesions 31. On the other hand, RNNs are specialized in processing sequential data such as speech recognition and image captioning 32. CRNNs combine the features of CNNs and RNNs and have been shown to be useful for assessing medical images by extracting spatial and temporal features from time-series medical imaging or image sequences 33. CRNNs have been used to differentiate between Parkinson's disease and healthy controls in multi-channel EEG signals 34 and to automatically diagnose Alzheimer's disease-related brain diseases from resting-state functional magnetic resonance imaging data 35. In addition, ViT-GRUs integrate the strengths of ViT and GRU, and can capture dynamic changes across images in longer time series, allowing for a better understanding of long-range temporal information 36, 37.

Previous studies have used machine learning models to analyze AV access sounds and predict AV access dysfunction. Grochowina et al. 38 introduced a prototype device for monitoring AVF using a supervised machine learning technique. Phono-angiograms were collected from 38 patients undergoing chronic HD, and features were extracted to create vectors for a decision-making algorithm. The results demonstrated a commendable accuracy of 81% for classifying acoustic signals into correct categories, which was confirmed by medical professionals at the dialysis center. In addition, Ota et al. 13 collected AVF sounds from 20 patients, converted them into spectrograms, and subsequently classified them into five distinct sound types using a CNN learning model. The accuracy ranged from 0.70 to 0.93, and the AUROC values ranged from 0.75 to 0.92. Despite the lack of direct correlation between sound type classification and AV access dysfunction, their results illustrated the potential of using a deep learning model to create an objective tool for the analysis of AV access sounds. Furthermore, Park et al. 14 demonstrated the feasibility of using deep learning-based analysis to predict significant AVF stenosis. They recruited 40 patients with dysfunctional AVF and recorded their AVF sounds before and after percutaneous transluminal angiography. The deep convolutional neural network models based on Mel spectrograms, especially ResNet50, demonstrated exceptional performance in predicting AVF stenosis of ≥ 50%, achieving AUROC values ranging from 0.70 to 0.99. However, the test data in that study were generated using synthetic techniques, and the models' generalizability to new patients was not assessed. In our study, we evaluated our models using distinct patients who had not been previously encountered by the model, providing a more accurate simulation of real-world clinical application.

This study has some limitations. First, we could not ascertain whether the individuals who did not undergo vascular intervention procedures had AV access stenosis, despite their AV access function being clinically adequate for HD. In addition, other associated causes of AV access dysfunction, such as central vein stenosis, may not be discriminated by the audio recordings, given that auscultation is difficult for detecting the presence of central vein stenosis. Second, the deep learning models could not differentiate the specific site of stenosis requiring a vascular intervention, such as an arterial site or venous site. Third, the study was limited to a single dialysis center, resulting in a relatively small sample size. Fourth, we did not collect audio recordings after vascular intervention as data for non-dysfunctional AV access when building the deep learning mode. Including this data could potentially enhance the model's performance. Lastly, although efforts were made to reduce noise by applying frequency-dependent filtering and trimming the initial and final 2 seconds of audio recordings, complete elimination of background noise during the recordings could not be achieved.

In conclusion, the CNN model based on Mel spectrograms outperformed the CRNN and ViT-GRU models in predicting malfunctioning AV access requiring vascular intervention within 10 days. This diagnostic approach holds promise as a non-invasive point-of-care tool for screening high-risk AV access in patients with HD.

## Figures and Tables

**Table 1:** The distribution of Patients and Audio Recordings in Training and Test Sets

Vascular intervention status	Training Set	Test set
Number of Patients		
Non-vascular intervention	43	12
Vascular intervention†	23	6
Total	66	18
Number of Audio Recordings		
Non-vascular intervention	196	83
Vascular intervention	111	47
Total	307	130

†: Patients with a history of vascular intervention were labeled as “vascular intervention” patients, and recordings which is in 10 days before were extracted as “vascular intervention recordings”. Conversely, patients who had never undergone vascular intervention were labeled as “non-vascular intervention” patients

**Table 2:** The architecture of the proposed CNN model.

Layer Description	Kernel Size	Shape	Channel
Input	-	40x369	3
Conv2D	8x20	33x350	64
ReLU Activation	-	33x350	64
MaxPool2D	2x2	16x175	64
Conv2D	4x10	13x166	64
ReLU activation	-	13x166	64
MaxPool2D	2x2	6x83	64
Flatten	-	31872	-
Fully connected	-	1	-
Sigmoid activation	-	1	-

Abbreviation: Conv2D, 2-dimentional convolution; ReLU, Rectified Linear Unit; MaxPool2D, 2-dimentional max pooling

**Table 3:** The architecture of the proposed CRNN model.

Layer Description	Kernel Size	Shape	Channel
Input	-	40x369	3
Conv2D	8x20	33x350	64
ReLU Activation	-	-	-
MaxPool2D	2x2	16x175	64
Conv2D	4x10	13x166	64
ReLU activation	-	-	-
MaxPool2D	2x2	6x83	64
Permute & Flatten	-	83x384	-
Bi-GRU(128 units)	-	83x256	-
Flatten	-	21248	-
Fully connected	-	1	-
Sigmoid activation	-	1	-

Abbreviation: Conv2D, 2-dimentional convolution; ReLU, Rectified Linear Unit; MaxPool2D, 2-dimentional max pooling; Bi-GRU, bidirectional gate recurrent unit

**Table 4:** The architecture of the proposed ViT-GRU model.

Layer Description	Operation Details	Shape	Repeat Block
Input	-	40x369x3	
Extract Patches^†^	-	366x192	
Patch Embedding	-	366x128	
LN	-	366x128	Repeat the block 2 times
MSA (8 heads)	0.1 dropout rate	366x128	
Residual connect		366x128	
LN	-	366x128	
MLP (2 layers)	GELU activation	366x128	
Residual connect		366x128	
LN	-	366x128	
Bi-GRU	128 units	366x256	
Flatten		93696	
Dropout	0.5 dropout rate	93696	
Fully connected	GELU activation	128	
Fully connected	Sigmoid activation	1	

Abbreviation: LN, layer normalization; MSA (8 heads), multi-headed self-attention with 8 heads; MLP (2 layers), multi-layer perceptron with 2 layers; GELU, gaussian error linear units; Bi-GRU, bidirectional gate recurrent unit †: Patches were extracted with the size of 8 and the stride of 6. The size of the input data was 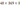. Each patch has the size of 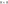, and the patches extracted from the 3 channels are concatenated and flattened. This results in the size of 192 of each patch, which can be calculated as: 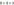. Totally, 366 patches were obtained, which can be calculated as: 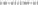.

**Table 5:** The results of the deep learning models based on out-of-fold predictions.

Metrics	CNN	CRNN	ViT-GRU
Accuracy	0.7362	0.7459	0.7720
Sensitivity	0.7928	0.7568	0.7838
Precision	0.6027	0.6222	0.6541
F1 Score	0.6848	0.6829	0.7131
AUC	0.7484	0.6723	0.7745

Abbreviation: CNN, Convolutional Neural Network; CRNN, Convolutional recurrent neural network; ViT-GRU, Vision Transformer and Gate Recurrent Unit; AUC, Area Under the receiver operating characteristic Curve

**Table 6:** The results of the deep learning models in the test set.

Metrics	CNN	CRNN	ViT-GRU
Accuracy	0.7538	0.7000	0.5923
Sensitivity	0.8085	0.7021	0.6170
Precision	0.6230	0.5690	0.4531
F1 Score	0.7037	0.6286	0.5225
AUC	0.7112	0.6381	0.5977

Abbreviation: CNN, Convolutional Neural Network; CRNN, Convolutional recurrent neural network; ViT-GRU, Vision Transformer and Gate Recurrent Unit; AUC, Area Under the receiver operating characteristic Curve
